# Rapid response systems in acute hospital care

**DOI:** 10.4103/1817-1737.58952

**Published:** 2010

**Authors:** Saad Al-Qahtani, Hasan M. Al-Dorzi

**Affiliations:** *Department of Intensive Care Unit, King Saud Bin Abdulaziz University for Health Sciences, Riyadh, Saudi Arabia*; 1 *Department of Intensive Care Unit, King Abdulaziz Medical City, Riyadh, Central Region, Saudi Arabia*

## Rationale for Rapid Response Systems

Medical emergencies frequently occur in hospitalized patients.[1–5] They include unplanned admission to the intensive care unit (ICU), in-hospital cardiac arrest and death. These events do not happen without warning. Several studies indicate that almost all critical inpatient events are preceded by warning signs for an average of 6–8 hours.[3,6,7] Such warning signs include: change in vital signs (such as tachycardia, tachypnea, and hypotension), acute dyspnea, and change in level of consciousness.[2,6,7] Simultaneously, suboptimal patient care is common. Studies in the US,[1] Canada,[8] Australia,[9] and the UK[10] estimate that adverse events occur in 10% of hospitalized patients with a mortality rate of 5–8%,[8,9] half of which are judged to be preventable.[8] Another study found that suboptimal care occurred in 54% of the hospitalized patients who required ICU admission with an ICU mortality of 48%; almost twice the mortality of well-managed patients.[10] This contributes to the failure to rescue at-risk hospitalized patients.

Failure to rescue patients in a healthcare facility is due to the following problems: failure of organization, lack of knowledge, failure to appreciate clinical urgency, lack of supervision, and failure to seek advice.[10] Should at-risk patients receive early intervention, in the form of better assessment and aggressive resuscitation, acute decompensation may be corrected before critical deterioration leading to improved outcome. This has triggered initiatives to improve the quality of care of acutely ill in-hospital patients. Bringing intensive care expertise to any acutely ill patient irrespective of location within the hospital, envisioned as ‘critical care without walls’ is one of these initiatives.[11] This is reflected by the increasing implementation of Rapid Response Systems (RRSs), variably named as medical emergency teams (METs) in Australia, rapid response teams (RRTs) in the US, critical care response teams (CCRTs) in Canada, and critical care outreach teams in the UK.[12]

The most common RRS, is a team of clinicians who bring critical care expertise to the bedside outside the ICU with the aim of intervening in the hours when patients show first sign of deterioration, thus, averting critical illness and cardiac or respiratory arrest.[12,13] The RRT is based on the notion of early and rapid intervention and is originally inspired by the management strategies of severe trauma, which included two key elements: the early detection of deterioration coupled to a rapid response. More recently, deployment of such teams was one of the main interventions recommended by the Institute for Healthcare Improvement in its ‘100,000 Lives Campaign’ that was launched in 2005.[13] Since then, thousands of RRTs have been instituted in North America and worldwide.[13]

## Components of the RRS

All RRSs are made up of at least four essential components.[12–14] The following is a description of these components:

An afferent limb which consists of ward healthcare givers who would recognize a deteriorating patient and activate the RRT.[12–14] This component is crucial as it links the actual team with the at-risk patient. Ward healthcare givers, such as, nurses and respiratory therapists, should be educated on the early signs of deterioration and the importance of early intervention. It should be noted that many healthcare facilities allow family members to activate the team.

An efferent limb, which is the actual team,[12–14] its composition depends entirely upon institutional goals, constraints, and resources. Hence, it can be either nurse- or physician-led and usually includes a respiratory therapist.[12–14] If nurse-led, the nurse usually has critical care experience and would request physician assistance if needed. When physician-led, the physician can be a hospitalist, a critical care fellow, an intensivist or an emergency physician. Regardless of the team composition, it should have all of the following: (a) ability and authority to prescribe medications; (b) advanced airway management skills, advanced cardiac life support certification is a must; (c) capability to establish central venous access; and (d) ability to provide an ICU level of care at the bedside.[12–14] Other key roles include transferring patients to ICU if needed and educating the ward staff.[12–14] Frequently, the team members carry with them medications and specialized equipment[14] to facilitate the timely treatment of the deteriorating patient. An essential requirement for the RRT success is building a friendly relationship with the afferent limb members. The notion of being ready to help at all times and the acceptance of soft activations should be part of every day job.

An administrative limb, which oversees all system components, empowers the team to be able to function and provides the needed resources.[12–14] Support from hospital administration is crucial for both team success and durability.

A quality improvement limb, which periodically reviews RRT activations, provides feedback on team function,[12–14] and monitors certain quality indicators such as staff satisfaction, monthly cardiac arrests occurring outside the ICU, ICU utilization, and annual hospital deaths per 1,000 discharges.[12,14]

## Activation of the Rapid Response Team

To early recognize the deteriorating patient in hospital wards and to facilitate the activation of the RRT by the afferent limb, the physiologic parameters reflecting or preceding clinical deterioration have been identified and used to formulate track and trigger warning systems. These are either single- or multiple-parameter systems, or aggregate weighted scoring systems.[15] The physiological parameters commonly used in single-parameter systems for RRT activation[13,14,16–18] are shown in Table 1

**Table 1:** Indications for rapid response team activation

• Acute change in heart rate	<40 or >130 beats per minute
• Acute change in systolic blood pressure	<90 mmHg or >200 mm Hg
• Acute change in respiratory rate	<8 or >30 per minute
• Acute change in saturation	<90% despite O_2_
• Acute change in conscious state	e.g., sudden fall in Glascow coma scale of >2 points
• Acute change in urinary output	<50 ml in 4 hours
• Repeated or prolonged seizures	

Multiple-parameter systems involve more than one criterion being met for the system activation and are rarely used.[15] The aggregate weighted scoring systems assign weighted scores to certain physiological parameters such as the heart rate, respiratory rate and body temperature, and compare the aggregate score to a predefined trigger threshold. Aggregate scoring systems are mainly used in the UK hospitals and most are based on modifications of the Early Warning Score, developed by Morgan *et al*.[15,19]

A systemic review of physiological track and trigger warning systems used to identify patients at risk showed that the commonly used warning systems had little reliability, validity, and utility.[15] This suggests that they should be used as adjuncts to clinical judgment as many at-risk patients will be missed if ward staff only relies on such objective criteria.[15] Hence, including a subjective activation criterion, such as serious concern or worry about a patient's condition is reasonable.[14] This empowers the ward staff to act upon their previous experience and clinical intuition in the absence of the above physiologic abnormalities.[14]

When a patient has any of the above criteria, the afferent limb members would activate the RRT either by overhead announcement or a dedicated pager or portable phone.[14] The RRT is expected to reach the at-risk patient within 15 minutes. Clear communication between the activating healthcare giver and RRT is important and hence communication using the situation-background-assessment-recommendation (SBAR) technique is recommended.[20] Having medical records and recent laboratory results readily available to the RRT facilitates prompt and optimal assessment of the situation. Documentation of the details of RRT encounter including assessments and recommendations is essential and should be part of the patient's medical record. RRT communication with the patient's attending physician or designee is also beneficial.

## The Evidence for RRS

The deployment of RRS in hospitals appears to be intuitive as it is inherently associated with better care, which is the goal of all healthcare givers. Several studies have evaluated the effectiveness of RRT. The majority of these studies are nonrandomized, before-and-after trials. Some of these studies suggested an outcome benefit in terms of reduced deaths, cardiac arrests, hospital length of stay, ICU length of stay, and cost.[16–18,21] For example, Buist *et al.* reported a reduction in unexpected death in hospital from 3.77 to 2.05 per 1,000 hospital admissions after implementation of MET.[16] The mortality from in-hospital cardiac arrest decreased in parallel from 77% to 56%[16] Similarly, Bellomo *et al.* showed that after implementation of MET, there were reductions in cardiac arrests by 65% (*P* = 0.001), deaths from cardiac arrest by 56% (*P* = 0.005), duration of ICU stay post arrest by 80% (*P* = 0.001), and inpatient deaths by 25% (*P* = 0.004).[17] Similar findings have been seen in pediatric patients.[21] In a cohort before-and-after study, implementation of an RRT was associated with a statistically significant reduction in hospital-wide monthly mortality rate by 18% and code rate outside of the pediatric ICU setting by 71%.[21] On the other hand, other studies showed neutral or negative effects of RRS implementation.[22–28] Bristow *et al.* evaluated the outcomes of patients at three hospital, one of which introduced MET, and found no significant difference in the rates of cardiac arrest or total deaths between the three hospitals.[22] Chan and colleagues prospectively assessed pre- and post-RRT outcomes in 24,193 and 24,978 adult patient admissions respectively and found no significant reduction in hospital-wide code rates [adjusted odds ratio (OR) = 0.76; 95% confidence interval (CI), 0.57–1.01; *P* = 0.06), and mortality (3.22 *vs.* 3.09 per 100 admissions; adjusted OR = 0.95; 95% CI, 0.81–1.11; *P* = 0.52).[24] Jones and colleagues found a significant increase in hospital mortality in medical patients after MET implementation.[25] In a retrospective before-and-after MET review, Brilli *et al.* found no significant difference in the incidence of pediatric cardiopulmonary arrests (risk ratio= 0.39; 95% CI, 0–1.4, *P* = 0.11) or hospital mortality (0.43 *vs*. 0.24 per 1,000 nonICU admits; risk ratio 0.55; 95% CI, 0–2.1, *P* = 0.23).[27]

Up-to-date, there are two cluster randomized controlled trials that evaluated RRS in the management of hospitalized adult patients.[28,29] Priestley and colleagues matched and randomized 16 hospital wards of a single nonteaching hospital in England to have either criticalcare outreach or standard care. They found that critical care outreach was associated with a reduced in-hospital mortality (adjusted OR = 0.52; 95% CI, 0.32–0.85) and with an increased mean length of stay (hazard ratio = 0.91; 95% CI, 0.84–0.99).[28] The other trial by Hillman and colleagues was done in 23 public hospitals across Australia and New Zealand and is known as the MERIT trial.[29] After two-month baseline period, 12 hospitals introduced METs (experimental hospitals) and 11 hospitals continued the usual management of patients using cardiac arrest teams (control hospitals) for four months. This was followed by six-month study period. Results showed that both groups of hospitals had a statistically significant 30% reduction in mortality compared to the baseline period. Moreover, the trial found no differences in the incidence of cardiac arrests, unplanned ICU admissions and unexpected deaths in the two groups of hospitals at the end of the intervention period.[29] Despite its robust design (cluster randomization), number of issues may have contributed to the trial inability to show a significant effect of METs.[12,30] These included: (a) incomplete and inconsistent implementation of METs in the experimental hospitals, as METs were activated for only 30% of patients who had physiologic deterioration according to MET activation criteria;[12,29,30] (b) possible implementation of RRSs by the control hospitals, which was not accounted for. This may have occurred as many of the hospitals were teaching hospitals;[12,29,30] (c) insufficient monitoring of relevant physiological variables in general ward patients;[12,30] and (d) low experimental power to detect improvements by MET because there were fewer events than expected and greater inter-institutional variability in event rates than anticipated when the trial was designed.[12,30] The latter point required the participation of higher number of hospitals to detect difference in outcomes.[12,30]

More recently, a systemic review of two randomized controlled trials, 16 uncontrolled before and after studies, three quasi-experimental studies, one controlled before and after study and one post-only controlled study, all done between 1996 to 2004, showed mixed results with respect to the following outcomes: mortality, cardiac arrest, unplanned critical care admissions from wards, length of stay, and critical care readmission rates. This suggested that the evidence for RRSs on improving the outcomes of hospitalized patients remains inconclusive.[31] Another systemic review showed that when comparing RRSs to control, the pooled relative risk for hospital mortality was 0.76 (95% CI, 0.39–1.48) in the same two randomized trials and 0.87 (95% CI, 0.73–1.04) in five observational studies. In addition, the pooled relative risk for cardiac arrest was 0.94 (95% CI, 0.79–1.13) in one of the randomized studies and 0.70 (95% CI, 0.56–0.92) in four observational studies, and for unanticipated ICU admissions was 0.84 (95% CI, 0.55–1.26) in four observational studies.[32] The authors concluded that the evidence that RRSs are associated with a reduction in hospital mortality and cardiac arrest rates is weak and that limitations in the quality of the original studies, the wide confidence intervals, and the presence of heterogeneity limited their ability to conclude that RRSs are effective interventions.

Obviously, research evaluating the effectiveness of RRS faces multiple challenges and difficulties.[33] Most of the studies done have been observational, nonrandomized and had before-and-after design. Before-and-after studies lack rigor and generalizability.[33] Moreover, the magnitude of the effect of a RRS may be influenced by the team structure, which is variable among hospitals and depends on institutional policies and available resources. It is also difficult to avoid the Hawthorne effect.[33] This may improve the care of control patients and reduce the differences in outcomes. Finally, cluster randomization of hospitals, which is the ideal methodology for RRS evaluation, requires the participation of large numbers of centers, which is difficult.[33]

## Conclusions

In summary, RRSs take the skills and expertise of the critical care team beyond the walls of the ICU within minutes to the bedside of deteriorating patients, whose condition may well progress to cardiac or respiratory arrest. RRSs would stabilize patients, prevent development of critical illness or cardiopulmonary arrest and contribute to the optimization of the care of other patients through education of healthcare givers working in the general medical and surgical wards. Their implementation requires significant resources and involves a change in the culture of healthcare provision. Although their merits look obvious and thus their deployment in hospitals seems to be intuitive, the available evidence for their effectiveness in improving the outcomes of such patients is weak and of suboptimal quality. Whether they should become the standard of acute hospital care needs to be answered.
